# Agri-Food and Food Waste Lignocellulosic Materials for Lipase Immobilization as a Sustainable Source of Enzyme Support—A Comparative Study

**DOI:** 10.3390/foods13233759

**Published:** 2024-11-24

**Authors:** Bartłomiej Zieniuk, Jolanta Małajowicz, Karina Jasińska, Katarzyna Wierzchowska, Şuheda Uğur, Agata Fabiszewska

**Affiliations:** Department of Chemistry, Institute of Food Sciences, Warsaw University of Life Sciences-SGGW, Nowoursynowska 159c, 02-776 Warsaw, Poland; bartlomiej_zieniuk@sggw.edu.pl (B.Z.); jolanta_malajowicz@sggw.edu.pl (J.M.); karina_jasinska@sggw.edu.pl (K.J.); katarzyna_wierzchowska1@sggw.edu.pl (K.W.); suheda_ugur@sggw.edu.pl (Ş.U.)

**Keywords:** buckwheat husk, loofah sponge, organic supports, pea hull, Sustine^®^ 121, *Yarrowia lipolytica*, yerba mate

## Abstract

Enzyme immobilization is a crucial method in biotechnology and organic chemistry that significantly improves the stability, reusability, and overall effectiveness of enzymes across various applications. Lipases are one of the most frequently applied enzymes in food. The current study investigated the potential of utilizing selected agri-food and waste materials—buckwheat husks, pea hulls, loofah sponges, and yerba mate waste—as carriers for the immobilization of Sustine^®^ 121 lipase and *Yarrowia lipolytica* yeast biomass as whole-cell biocatalyst and lipase sources. Various lignocellulosic materials were pretreated through extraction processes, including Soxhlet extraction with hexane and ethanol, as well as alkaline and acid treatments for loofah sponges. The immobilization process involved adsorbing lipases or yeast cells onto the carriers and then evaluating their hydrolytic and synthetic activities. Preparations’ activities evaluation revealed that alkaline-pretreated loofah sponge yielded the highest hydrolytic activity (0.022 U/mg), while yerba mate leaves under brewing conditions demonstrated superior synthetic activity (0.51 U/mg). The findings underscore the potential of lignocellulosic materials from the agri-food industry as effective supports for enzyme immobilization, emphasizing the importance of material selection and pretreatment methods in optimizing enzymatic performance through giving an example of circular economy application in food processing and waste management.

## 1. Introduction

Enzyme immobilization is a pivotal technique in biotechnology that enhances the stability, reusability, and overall efficiency of enzymes in various applications, particularly in biocatalysis. This process involves attaching enzymes to solid supports, allowing for easier separation and recovery of the enzyme after the reaction, which is crucial for industrial applications where cost-effectiveness and sustainability are paramount [1,2]. The immobilization of enzymes can significantly improve their operational stability and facilitate their use in continuous processes, thereby increasing productivity and reducing waste. Several methods are employed for enzyme immobilization, each with distinct advantages and limitations. The primary techniques include adsorption, covalent binding, entrapment, and cross-linking [3].

The choice of support material is also crucial for successful enzyme immobilization. Immobilization supports can be divided into inorganic and organic. Inorganic materials offer stability and inertness, but their shape possibilities are limited and prone to abrasion when stirred. Widely used materials for enzyme immobilization include silica, inorganic oxides, mineral materials, and carbon-based materials. Innovative inorganic carriers such as magnetic particles, carbon nanotubes, graphene, and its oxide provide enhanced options for enzyme immobilization [4]. In the case of organic carriers, there are biopolymers and synthetic polymers. In particular, biopolymers (e.g., alginates, chitosan, cellulose, and derivatives) have unique properties (biocompatibility, biodegradability, non-toxicity) and exceptional affinity for proteins, making them an excellent choice for enzyme immobilization. The availability of reactive functional groups in the biopolymer structure, mainly hydroxyl, as well as ammonium and carbonyl, sets them apart from inorganic materials and enables a direct reaction between the enzyme and the matrix. The flexible nature of biopolymers allows for the creation of various shapes and facilitates the modification of their surface [4,5,6,7]. Furthermore, due to their unique properties, there is a growing interest in using lignocellulosic materials as support [8]. Lignocellulosic materials, derived from plant biomass, offer several advantages as enzyme supports. These are widely available as agricultural waste, making them a low-cost option for enzyme immobilization [7]. Secondly, utilizing lignocellulosic waste contributes to waste reduction and promotes a circular economy, aligning with sustainability goals [1]. Furthermore, such materials possess a high surface area and porosity, which can enhance enzyme loading and activity, and last but not least, lignocellulosic supports are generally biocompatible, being derived from natural sources, making them suitable for various biotechnological applications [9]. Despite their advantages, lignocellulosic materials have challenges associated with using them for enzyme immobilization. These include potential variability in material properties, the need for pre-treatment to enhance accessibility, and the possibility of reduced enzyme activity due to steric hindrances [10].

Hydrolytic enzymes, including amylases, proteases, and lipases, are used to improve food quality. The latter find their application in flavor enhancement in cheese and meat products and fat function modification by interesterification [11].

The study aimed to assess the possibility of using selected agri-food and waste materials, namely buckwheat husks, pea hulls, loofah sponges, and yerba mate waste, as carriers for immobilization lipases: Sustine^®^ 121 commercial lipase solution (previously called Lipozyme^®^ TL 100 L) and *Yarrowia lipolytica* yeast biomass (source of whole-cell biocatalysts with lipolytic activity) and to assess the hydrolytic and synthetic activity of the obtained immobilized biocatalysts. The research is an example of sustainable solutions in the food industry. The presented study brings an example of the valorization of agri-food industry waste and interesting applications of food raw materials.

## 2. Materials and Methods

### 2.1. Materials

The materials used in this study were lignocellulosic waste materials of agri-food origin, including pea (*Pisum sativum*) hulls, buckwheat (*Fagopyrum tataricum*) husks, loofah (*Luffa cylindrica*) sponge, and commercially available dried yerba mate (*Ilex paraguariensis*) leaves (Yerba Mate Yacuy TERERE Pure Leaf Premium, Brazil).

Sustine^®^ 121 (Lipozyme^®^ TL 100 L) liquid lipase from *Thermomyces lanuginosus* was kindly donated by Novozymes (Bagsværd, Denmark). All chemical reagents and solvents were purchased from Sigma-Aldrich (Poznań, Poland) and Avantor Performance Materials Poland S.A. (Gliwice, Poland).

*Y. lipolytica* KKP 379 from the Collection of Industrial Microorganisms of the Prof. Waclaw Dabrowski Institute of Agricultural and Food Biotechnology—State Research Institute (Warsaw, Poland) was also used in the experiments.

### 2.2. Pretreatment of Lignocellulosic Materials

**Figure 1:**
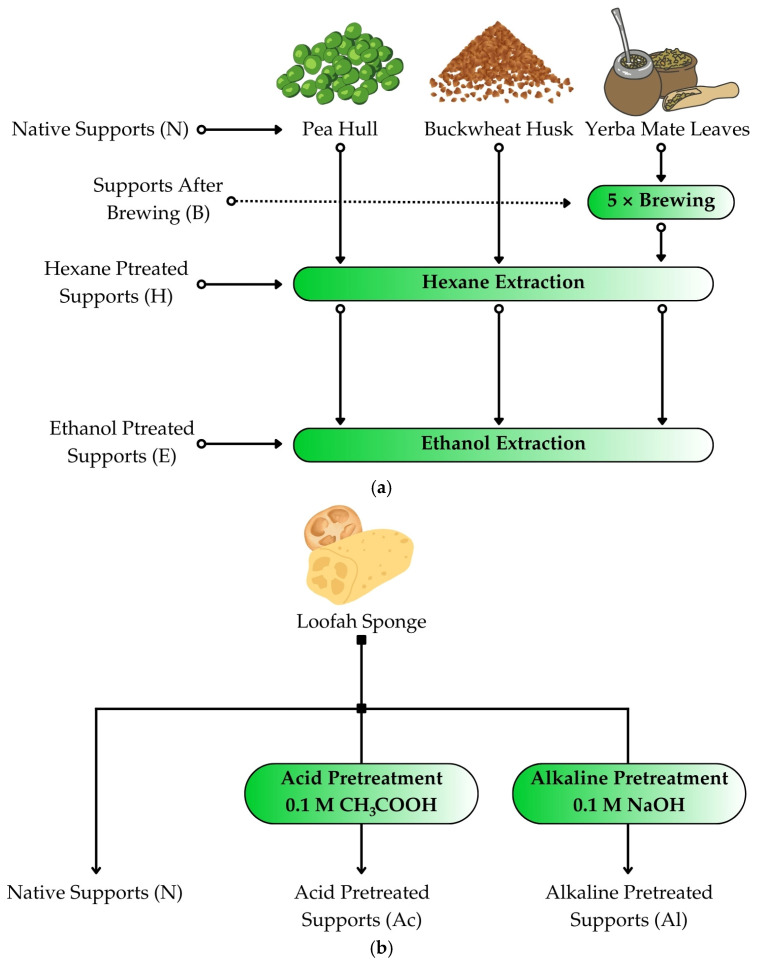
Graphical representation of the pretreatment process of lignocellulosic materials, i.e., (**a**) pea hull, buckwheat husk, or yerba mate leaves, and (**b**) loofah sponge.

The lignocellulosic carriers, i.e., pea hulls, buckwheat husks, and yerba mate leaves were prepared by weighing 10 g of each material and grinding them in a coffee grinder (Esperanza EDC Poterek Sp. J., Ożarów Mazowiecki, Poland). The crushed materials were then subjected to extraction in a Soxhlet apparatus. For pea hulls and buckwheat husks, the materials were divided into three groups: native carriers without extraction (N), carriers extracted with hexane (H), and carriers extracted with hexane and then with ethanol (HE). As for yerba mate, due to its high content of active substances, it was divided into four groups: native carriers without brewing or extraction (N), carriers brewed five times (B), brewed carriers extracted with hexane (BH), and brewed carriers extracted with hexane and ethanol (BHE) (Figure 1a). Yerba mate was brewed by adding boiling water to the dried material, covering it with a vessel and allowing it to cool. The water was then drained, and the brewing process was repeated another four times. After brewing, the material was dried and ground in a coffee grinder.

The Soxhlet extraction process involved wrapping 10 g of ground raw materials in filter paper and placing them in the extractor chamber. Then, depending on the selected solvent, 150 mL of ethanol or hexane was added to the flask. Extraction continued until the extract was poured into the flask 12 times. After the extraction, the remaining solvent was evaporated from the flask using the Büchi Rotavapor R-200 (Büchi AG, Flawil, Switzerland).

A *Luffa cylindrica* sponge was cut into 1 × 1 cm^2^ cubes and then placed in crystallizers with distilled water (native support, N), 0.1 M sodium hydroxide (NaOH) solution (alkaline pretreatment, Al), or 0.1 M acetic acid (CH_3_COOH) solution (acid pretreatment, Ac; Figure 1b). The crystallizers were placed on magnetic stirrers with heating, and the solutions were heated to 80 °C for about 1 h while stirring constantly. After this time, the sponges were drained and washed three times with distilled water to a pH of about 7. Then, they were dried at 80 °C for 24 h until reaching a constant weight.

### 2.3. Elemental Compositions of Lignocellulosic Materials

The contents of total carbon, nitrogen, and sulfur were analyzed using dry combustion with the Vario MacroCube elemental analyzer (Elementar, Langenselbold, Germany).

### 2.4. Scanning Electron Microscopy (SEM)

The surface and morphology of the native supports and yeasts immobilized onto the loofah sponge were examined by an electron microscope (HITACHI TM 3000, Ramsey, NJ, USA). Before observation, the samples were dried under a vacuum and coated with a layer of gold (Cressington 108 Auto Sputter Coater, Cressington Scientific Instruments, Watford, UK). Micrographs were taken at a magnification of 400×, 600×, and 1000×.

### 2.5. Fourier Transform Infrared Spectroscopy (FTIR)

The FTIR spectra of all materials were recorded using a Nicolet iS5 ATR Thermo Scientific spectrometer equipped with a diamond crystal iD7 ATR sampling component (Thermo Fisher Scientific, Waltham, MA, USA). The measurements were taken in the range of 4000–600 cm^−1^.

### 2.6. Immobilization of Sustine^®^ 121

Sustine^®^ 121 was immobilized by adsorption onto different lignocellulosic materials without and after purification processes. It was carried out by weighing 1 g of each previously prepared carrier. The weighed materials were placed in round-bottom flasks, and then 1 mL of liquid lipase and 14 mL of distilled water were added and then agitated for 2 h. After that time, the supernatant from the flasks was filtered and collected to check the amount of enzyme adsorbed onto the carrier. The carriers with immobilized lipases were rinsed with distilled water, filtered, and placed on Petri dishes. The immobilized lipases were then dried at room temperature.

### 2.7. Immobilization of Yarrowia lipolytica Cells on Loofah Sponge

*Y. lipolytica* KKP 379 cells from Yeast Extract-Peptone-Glucose Agar slants were transferred into 100 mL of liquid YPG media. Microorganisms were cultured for 24 h at 28 °C in an IKA KS 4000 ic control shaker (IKA company, Konigswinter, Germany) at 140 rpm. Then, 1 mL of the 24 h yeast inoculum in the logarithmic phase of cells was transferred to a liquid YPG medium. Batch cultures were conducted for 120 h at 28 °C with shaking at 140 rpm.

Two experimental variants carried out the process of immobilizing the whole-cell catalyst, *Y. lipolytica* yeast cells. One gram of sterilized sponges was introduced into flasks with 100 mL of medium and in:

**Variant I:** A loofah sponge was introduced into the microbiological medium along with yeast inoculum and remained in the solution throughout the entire yeast growth cycle.

**Variant II:** The loofah sponge was introduced after the prior multiplication of cells—after a period of 48 h of cultivation.

The binding efficiency of yeast cells to the carrier was determined based on the difference in dry mass between the sponge with immobilized yeast cells and the native sponge present in the culture medium (control sample).

### 2.8. Analysis of the Optical Density of Yeast Cultures

One mL of the biomass suspension was centrifuged using the Eppendorf microcentrifuge, model 5418 (Carl Roth GmbH + Co. KG, Karlsruhe, Germany). The supernatant was carefully decanted, and the cells were suspended in 1 mL of distilled water. For such a prepared sample, the optical density (OD) was measured after its appropriate dilution using a spectrophotometer RayLeigh UV-1601 (BRAIC, Beijing, China) at a wavelength of 600 nm.

### 2.9. Determination of Yeast Cell Dry Weight

A comparison was conducted to assess the yeast cell dry weight between the control culture and the cultures with the addition of a loofah sponge. For this purpose, 10 mL of suspension was taken from the flasks and transferred to pre-weighed falcon tubes. Subsequently, the yeast cells were separated from the supernatant through centrifugation in an MPW centrifuge (MPW Med. Instruments, Warsaw, Poland) at a speed of 8000 rpm for 10 min. The supernatant was decanted from the biomass, and the yeast cells were dried until a constant mass was achieved in a drying oven at approximately 80 °C for about 24 h. Based on the difference in mass between the falcon with dry yeast cell mass and the empty falcon, the obtained dry cell weight was calculated. The determination was performed in two replicates, giving the result in g d.m./L.

### 2.10. Protein Content

Protein concentration was determined through spectrophotometry using the Lowry method, as outlined in Jasińska et al. [12]. The protein adsorbed onto the lignocellulosic materials was calculated based on the difference between the protein concentration in free lipase and the post-immobilization filtrates.

### 2.11. Hydrolytic Activity

A spectrophotometric method was employed to assess the hydrolytic activity of the obtained biocatalysts. Specifically, 25 mg of immobilized biocatalyst was suspended in 100 μL of distilled water, along with 25 μL of 0.3 mmol *p*-nitrophenyl laurate dissolved in 2 mL of heptane. The resulting mixture was stirred at 37 °C. After 15 min, the absorbance was measured at 410 nm using a UV–Vis spectrophotometer (RayLeigh UV-1601, BRAIC, Beijing, China). The unit of lipase enzymatic activity was 1 U, defined as the amount of enzyme that released one µmol of *p*-nitrophenol per minute under the assay conditions. The obtained hydrolytic activity and protein content results were also used to calculate the specific activities of immobilized biocatalysts [13].

### 2.12. Synthetic Activity

The effectiveness of the immobilized lipase was also assessed using a colorimetric synthetic method based on Jasińska et al. [12] protocol. The test involved a transesterification reaction between vinyl acetate (100 mM) and 1-butanol (100 mM) conducted in an Eppendorf tube in 1 mL of hexane, including 5 mg of immobilized lipase. The assay included preparing diluted samples, adding MBTH (3-methyl-2-benzothialinone) and H_4_FeNO_4_S_2_·12H_2_O solutions, and conducting colorimetric measurements at 595 nm. The unit of lipase synthetic activity was 1 U, indicating the amount of enzyme that converted 0.1 mmol of vinyl acetate into acetaldehyde per minute under the assay conditions. Similarly to Section 2.11, specific synthetic activities were also calculated.

### 2.13. Statistical Analysis

Statistical analysis was performed using Statistica 13.3 software (TIBCO Software Inc., Palo Alto, CA, USA). The results were analyzed using one-way analysis of variance (ANOVA) and Tukey’s post hoc test. The significance level was α = 0.05.

## 3. Results and Discussion

### 3.1. Application of Lignocellulosic Materials for Immobilization of Sustine^®^ 121

**Figure 2:**
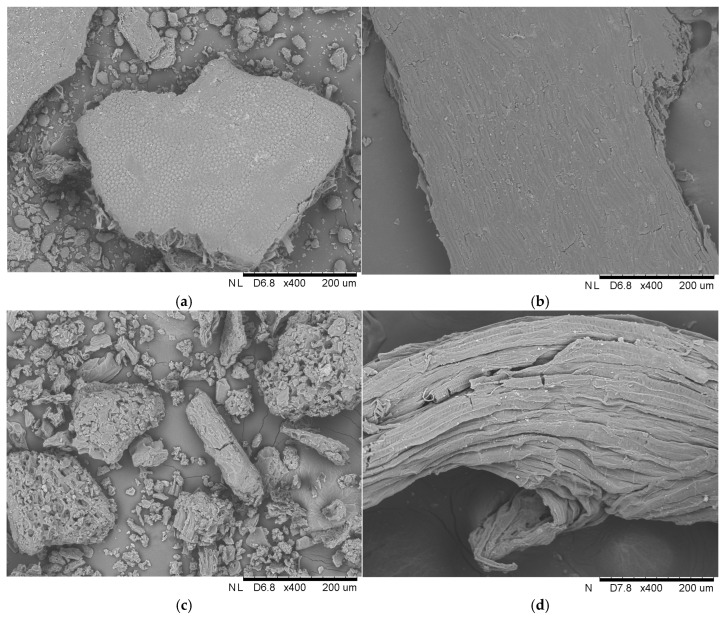

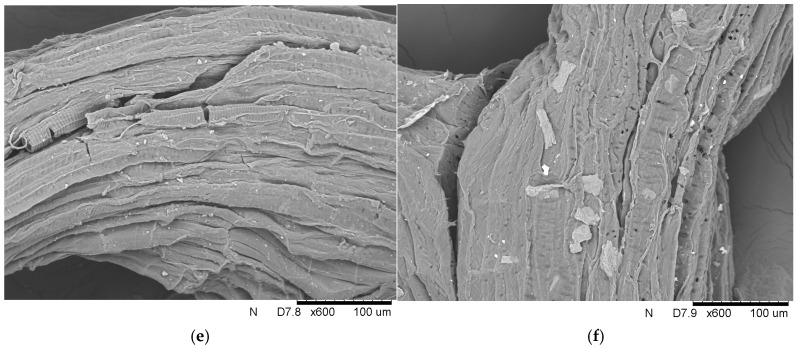
Scanning electron microphotographs of (**a**) pea hull, (**b**) buckwheat husk, (**c**) yerba mate leaves, and (**d**) loofah sponge at 400× magnification, and (**e**) and (**f**) loofah sponge at 600× magnification.

Efforts to repurpose lignocellulosic materials have increased due to the rising interest in sustainable practices. While the approach of the application of lignocellulosic waste as immobilization carriers may not be universally embraced as a global management strategy, it undeniably represents valuable efforts to explore innovative solutions for utilizing such waste. In the current study, four different raw materials were used for the immobilization of liquid lipase, i.e., pea hull, buckwheat husk, yerba mate leaves, and loofah sponge. The studies started with analyzing the surface of these materials with scanning electron microscopy (Figure 2).

The analysis revealed large particles with a smooth surface and no visible cavities in the pea hull and buckwheat husk. In contrast, the analysis of yerba mate clearly showed smaller and less organized fiber particles. Additionally, fragmented pieces of the carrier, with densely arranged cavities and characteristic jaggedness, were observed. Unlike the abovementioned materials, the loofah sponge was characterized by its porous and irregular structure, which is evident in the SEM image. The rough surface has distinct fibrous cords with visible grooves and microcracks. In addition, the fiber surface has bright, similar-sized spots, which, according to Guo et al. [14], were pectin and wax impurities. The network of fibers arranged in a multidirectional arrangement creates an elastic mesh with exceptional properties.

Each sample’s unique structural features contribute to its final possible application as an immobilization support. In the case of the luffa sponge, Chen et al. [15] revealed that the high porosity of the sponge combined with numerous pores and channels and high specific surface area facilitates the immobilization process. In the review article of Mohamad et al. [16], it was found that the support matrix must possess mesoporous material with a large surface area and numerous pores to achieve a higher enzyme loading per unit mass. Furthermore, the support should be characterized by the previously mentioned good porosity and large surface area, but also an appropriate hydrophobic/hydrophilic balance, which allows the enzyme to act with greater efficiency [17].

To confirm the effective immobilization of lipases onto *Luffa cylindrica* sponge, buckwheat husk, yerba mate leaves, and pea hulls, FTIR (Fourier Transform Infrared Spectroscopy) analysis was performed. The compiled FTIR spectra were placed in the supplementary materials and presented in Figures S1–S8. The spectra of the control samples—native sponge, buckwheat, yerba mate, and pea hulls—were compared with the spectrum of the pretreated carriers. Spectra of the sponge pretreated with acetic acid or sodium hydroxide were compared with the non-treated raw sponge (Figure S1). Treatment with NaOH or acetic acid influenced the intensity of frequency regions in terms of wave number 1020 cm^−1^–1000 cm^−1^ corresponding to the bending vibrations of the C=C bonds. Additionally, the spectrum of the sponge treated with the NaOH revealed the lower frequency of regions in terms of wave number 1870 cm^−1^–1540 cm^−1^ corresponding to the stretching vibrations of the C=O bonds. Similarly, non-treated native buckwheat husks, native yerba mate leaves, and native pea hulls were compared with hexane and ethanol-treated carriers (Figures S3, S5 and S7). Hexane treatment of buckwheat resulted in a significant decrease in the bands 2916 cm^−1^ and 2848 cm^−1^ stretching N-H in amines and C-H in alkenes (Figure S3). Few changes in spectra were observed for yerba mate (Figure S5). It seemed that treatment with solvents and brewing did not reveal changes in functional groups in the carrier. In the case of pea hulls, the disappearance of the band 1735 cm^−1^ corresponded to the stretching C=O bonds, which could not be seen for spectra of carriers treated with hexane and ethanol (Figure S7). The effect of support treatment on the activity of immobilized biocatalysts was, apart from the sponge, caused by the removal of selected support components and not by any specific change in the ratio of functional groups in the support.

FTIR analysis was performed for a carrier on which the enzyme lipase was immobilized (Figures S2, S4, S6 and S8) to investigate the presence of characteristic functional groups for proteins in immobilized systems. In the spectra of proteins, characteristic so-called amide bands are present, three of which are most important in analyzing the secondary structure of proteins. They are as follows: amide band, corresponding mainly to the stretching vibrations of the C=O bond (1700 cm^−1^–1600 cm^−1^), amide band, corresponding to the bending vibrations of the N-H bond, and the stretching vibrations of the C-N bond (1600 cm^−1^–1500 cm^−1^) and amide band, corresponding mainly to the stretching vibrations of the C-N bond and the bending vibrations of the N-H bond (approx. 1340 cm^−1^–1200 cm^−1^) [18]. In the carrier spectrum after the enzyme immobilization process, a peak with a frequency of 1735 cm^−1^ was visible, characteristic of C=O bonds responsible for binding the enzyme to the support. In the case of the sponge, an increase in the intensity of the bands 1730–1740 cm^−1^ was observed in the characteristics of proteins, but only for untreated carriers and sponges treated with acetic acid (Figure S4). For the buckwheat carrier, the intensity could be seen in bands 1600–1601 cm^−1^ for the hexane and ethanol-treated carrier (Figure S6). A similar observation was made for peas hulls. Regardless of the method of carrier preparation, an increase in the intensity of the bands in the wavenumber range 1630–1640 cm^−1^ was seen (Figure S8).

Understanding the composition of the materials used for immobilization is significant for choosing the right carrier. The elemental analysis of various supports used for the immobilization of Sustine^®^ 121 reveals significant differences in the chemical composition of each support material. The data presented in Table 1 include carbon, nitrogen, and sulfur percentage content across different pretreatment conditions.

The carbon content varies significantly among the supports, with yerba mate leaves showing the highest carbon percentage at approximately 50%, while pea hulls had lower carbon percentages of around 41%. Additionally, yerba mate leaves had also the highest nitrogen and sulfur levels. The pretreatment of pea hulls and buckwheat husks did not significantly change their elemental composition, but it had different effects on yerba mate leaves, especially in carbon content, which may affect enzyme activity after immobilization and could indicate the removal (extraction) of some substances present in this material. Rakocevic et al. [19] indicated that carbon and nitrogen levels depend on the cultivation environment, specifically monoculture and agroforestry. Carbon levels ranged from 46% to 47% while nitrogen levels ranged from approximately 1.15% to 1.45%. Zhang et al. [20] demonstrated that the carbon content in the loofah sponge was 48.35%. The elemental analysis of spent coffee grounds (SCG), used as an immobilization support, was accomplished by Girelli et al. [21]. Their research confirmed that a decrease in the percentage of specific elements is linked to processes such as protein extraction using water pretreatment (reduction in nitrogen content) or defatting with organic solvents (reduction in carbon content).

**Figure 3:**
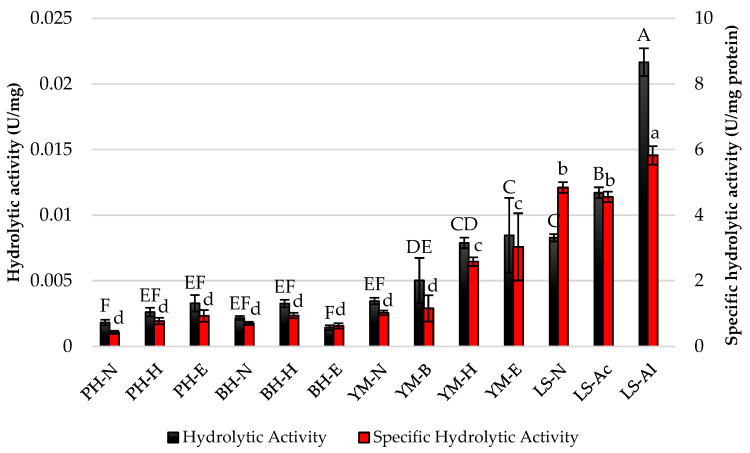
Hydrolytic and specific hydrolytic activities of Sustine^®^ 121 immobilized on different lignocellulosic materials. Error bars represent the standard deviation (SD) of three independent measurements. The values with the same uppercase (A–F) or lowercase letters (a–d) did not differ significantly (α = 0.05). Abbreviations: PH—pea hull, BH—buckwheat husk, YM—yerba mate, LS—loofah sponge, N—native support, B—brewing, H—hexane pretreatment, E—hexane and ethanol pretreatment, Ac—acid pretreatment, Al—alkaline pretreatment.

After the Sustine^®^ 121 lipase was immobilized onto previously prepared supports, hydrolytic and specific hydrolytic activities were assessed (Figure 3). The highest hydrolytic activity was observed with the alkaline-pretreated loofah sponge (LS-Al) at 0.022 U/mg, indicating its effectiveness as a support material for Sustine^®^ 121. Specific hydrolytic activity also peaked with LS-Al at 5.82 U/mg protein, demonstrating a strong correlation between the type of lignocellulosic material and enzyme performance. Interestingly, the various pretreatments significantly influenced both hydrolytic and specific hydrolytic activities, suggesting that optimization of support materials can enhance enzyme efficacy.

In the case of natural fibers, alkaline treatment is one of the frequently used chemical methods. During the process, the OH groups on the fiber surface react and dissolve by transforming into cellulose alkoxides, which improves the fiber’s moisture resistance and increases the fiber’s adhesive properties. Acid treatment enhances cellulose structures and improves their surface roughness, including by eliminating lignin, wax, and pectin, which was confirmed, among others, by treating the *Furcraea foetida* fibers with acetic acid [22].

**Figure 4:**
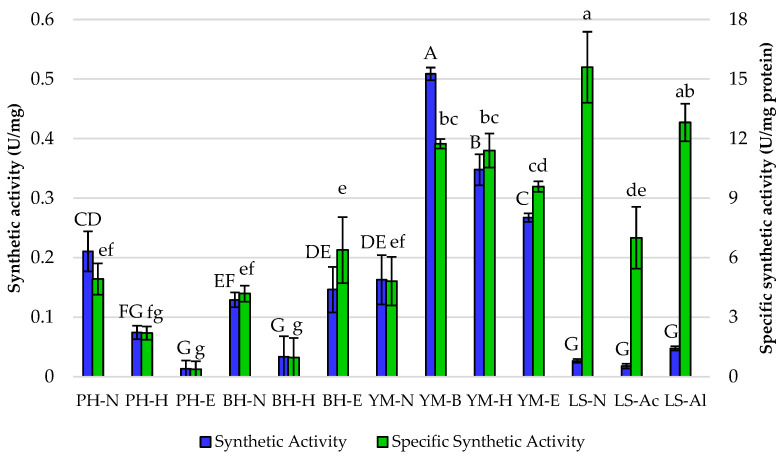
Synthetic and specific synthetic activities of Sustine^®^ 121 immobilized on different lignocellulosic materials. Error bars represent the standard deviation (SD) of three independent measurements. The values with the same uppercase (A–G) or lowercase letters (a–g) did not differ significantly (α = 0.05). Abbreviations: PH—pea hull, BH—buckwheat husk, YM—yerba mate, LS—loofah sponge, N—native support, B—brewing, H—hexane pretreatment, E—hexane and ethanol pretreatment, Ac—acid pretreatment, Al—alkaline pretreatment.

Synthetic and specific synthetic activities were also evaluated (Figure 4). The highest synthetic activity was observed with yerba mate under brewing conditions (YM-B) at 0.51 U/mg, suggesting that this substrate and these conditions may be particularly favorable for the enzyme’s performance. In contrast, the loofah sponge showed low synthetic activity across all pretreatments, particularly with native support (LS-N) at 0.03 U/mg. In the case of specific synthetic activity, i.e., the lipase activity per milligram of total protein, the highest activity was recorded for LS-N at 15.59 U/mg, which suggests that loofah sponge, despite lower immobilization yields, allowed for efficient enzyme action. The significant specific synthetic activity was also observed for Sustine^®^ 121 immobilized onto the LS-Al (12.81 U/mg), indicating one of the best performances of this preparation among all tested in terms of both hydrolytic and synthetic properties.

Lipases YM-B and YM-H also showed remarkable specific synthetic activities at 11.74 and 11.40 U/mg, respectively. Comparing only yerba mate as a support, the highest specific synthetic activity was observed for YM-B, indicating that brewing effectively enhances the enzyme’s performance. Thus, steeping the yerba mate leaves in hot water allowed for extracting soluble components and changed the availability of cellulose, making it more accessible for enzymatic action. The decrease in specific synthetic activity observed after hexane and ethanol treatments highlights the importance of carefully controlling pretreatment conditions. Given the observed specific synthetic activities, brewing alone is likely sufficient to achieve the highest possible activity for lipase immobilization on yerba mate leaves, and further pretreatments did not provide additional benefits.

Interestingly, the relatively high activity of lipase immobilized onto alkaline pretreated material may be due to the effective removal of lignin from lignocellulosic materials through sodium hydroxide solution. A decrease in the lignin content was acknowledged by Zheng et al. [23], where the authors effectively removed lignin from wheat straw. In turn, acid pretreatment demonstrates high efficacy in removing hemicellulose but can lead to some degradation of cellulose at higher concentrations and temperatures, which are crucial for efficient enzyme immobilization. Jasińska et al. [13] demonstrated that removing the hemicellulose from SCG negatively influenced the activity of immobilized enzymes.

Moreover, hexane, ethanol, and brewing processes improved substrate accessibility for enzymatic assays. It was seen primarily for yerba mate leaves, where the activity of immobilized lipase increased after each process. So far, only one study has been conducted on the possibility of using yerba mate for lipase immobilization. Eversa^®^ Transform 2.0 lipase was immobilized onto the residual yerba mate sticks. In the study of Rigo et al. [24], this lignocellulosic material was first purified by alkaline pretreatment, and then lipase was covalently immobilized after activation with aminopropyltriethoxysilane/glutaraldehyde and sodium metaperiodate. Such prepared immobilized lipases exhibited good operational stability and the optimal temperature of 40 °C [24].

### 3.2. Immobilization of Y. lipolytica KKP 379 onto Loofah Sponge

**Figure 5:**
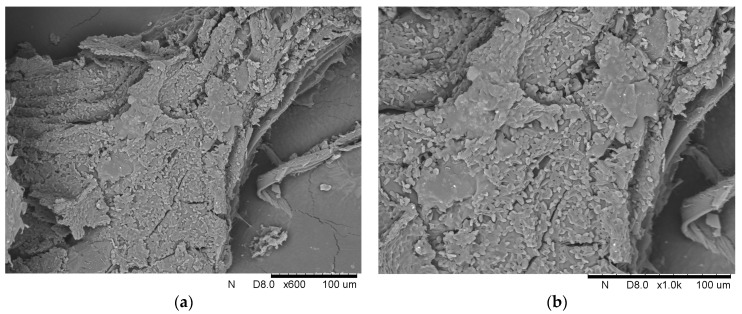
Scanning electron microphotographs *Y. lipolytica* cells immobilized onto a loofah sponge with the magnification of (**a**) 600× and (**b**) 1000×.

The second part of this work concerned using the loofah sponge to immobilize the yeast *Y. lipolytica*. Two different approaches for immobilization were compared. The first involved adding 1 g of loofah sponge at the beginning of the culture, while the second added 1 g after 48 h. This comparison aimed to determine the most effective strategy for such a process. In both variants, immobilization was thoughtfully confirmed using SEM photography, OD measurements, biomass yield, and analysis of the weight change of the loofah sponge after yeast culture. Figure 5. presents scanning electron microphotographs of *Y. lipolytica* cells immobilized onto a loofah sponge at magnifications of 600× and 1000×, which illustrate the arrangement of the cells in this immobilization matrix.

At both 600× and 1000× magnifications, the photographs reveal a detailed view of the yeast oval cells adhering to the porous surface of the loofah sponge. The photographs show clusters of cells, demonstrating their ability to form aggregates on the loofah fibers.

Several other studies have shown the possibility of using the loofah sponge in microbial and biotechnology applications.

Due to its unique structure and special physical and mechanical properties, the *Luffa* sponge was first used as a matrix for immobilizing algal cells as early as 1993 [25]. Its low density and, at the same time, very high porosity and proper pore volume distinguish it from other carriers for immobilizing cells by adhesion. *Luffa cylindrica* has also been successfully used as a matrix for the immobilization of actively growing *Aspergillus niger* cells secreting amylolytic enzymes, including α-amylase and glucoamylase [25]. It was also used as a carrier in the immobilization of *Streptomyces clavuligerus*, synthesizing clavulanic acid—a potent inhibitor of bacterial beta-lactamases [26]. Beher and Ray [27] used the sponge to immobilize yeast *Saccharomyces cerevisiae* during bio-ethanol production from sugarcane molasses. Liu et al. [28] presented the sustainable production of erythritol, a low-caloric sweetener, alongside the utilization of waste cooking oil, where loofah sponge was used to disperse oil, resulting in a 30% increase in erythritol production by *Y. lipolytica*. Moreover, Try et al. [29,30] explored solid-state fermentation of *Y. lipolytica* W29 and loofah sponge as a support in the production of γ-decalactones, well-known aroma compounds.

**Figure 6:**
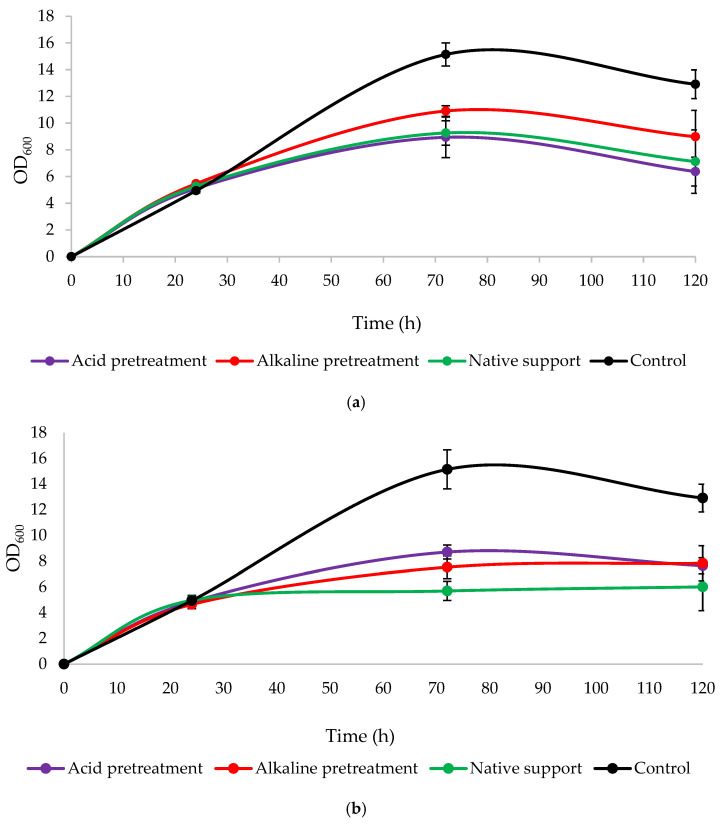
Changes in optical density (OD_600_) during 120 h culture of *Y. lipolytica* KKP 379 when: (**a**) 1 g of loofah sponge was added at the beginning of culture and (**b**) 1 g of loofah sponge was added after 48 h of culture.

Changes in the optical density (Figure 6) acknowledged yeast growth in the presence of a loofah sponge. The control cultures (without LS in the medium) in both variants had the highest OD_600_ values. Then, significant growth was observed across all variants, particularly in the alkaline pretreatment of LS and its addition at the beginning of the culture, reaching a peak of 10.905 at 72 h. The prolonging of the cultures resulted in a decline in OD_600_ in most cultures or their values remained constant. The lower OD_600_ values using the sponge relative to the control appear to confirm that this lignocellulosic material is an outstanding carrier for the immobilization of enzymes and whole microbial cells.

**Figure 7:**
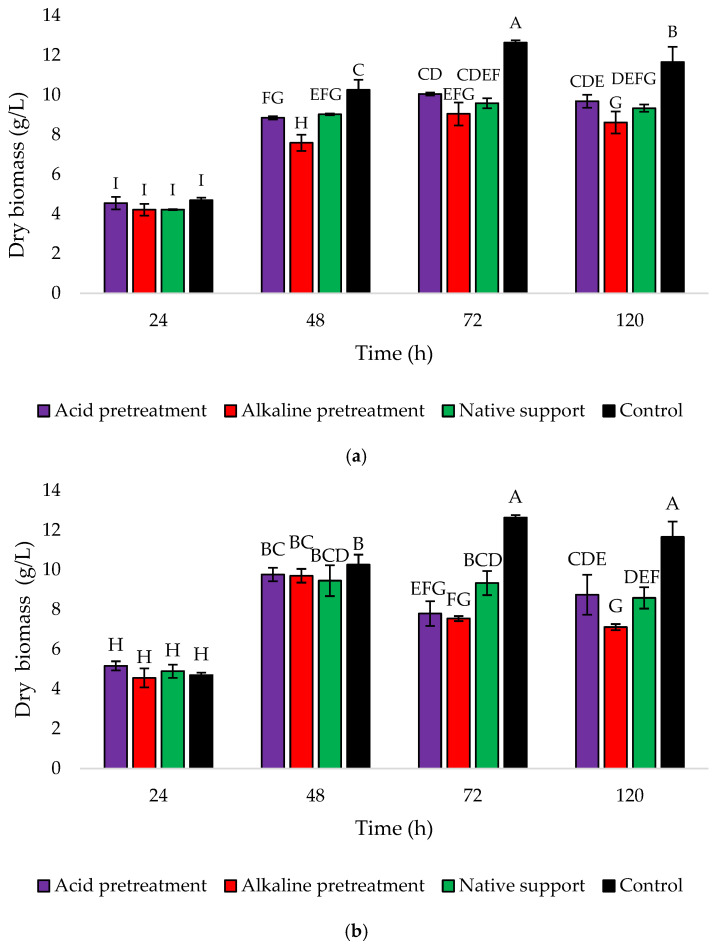
Changes in the yeast dry biomass (g/L) during 120 h culture of *Y. lipolytica* KKP 379 when: (**a**) 1 g of loofah sponge was added at the beginning of culture and (**b**) 1 g of loofah sponge was added after 48 h of culture. Error bars represent the standard deviation (SD) of three independent measurements. The values with the same uppercase letters (A–I) did not differ significantly (α = 0.05).

The findings were also confirmed through the analysis of dry biomass changes during 120 h cultures (Figure 7). At the 24 h mark, all treatments showed similar and relatively low biomass ranging from 4.21 to 4.69 g/L for Variant I. Then, there was a consistent increase in biomass until the maximum at the 72 h mark, followed by a slight decrease at the end of the culture period (Figure 7a). In the case of Variant II, after reaching a peak at 48 h, there was a noticeable decline in biomass at 72 h for both acid and alkaline pretreatments. The dry biomass of yeast cells in the medium with the acid-pretreated sponge dropped from 9.72 to 7.79 g/L, while with the alkaline-pretreated sponge from 9.70 to 7.75 g/L. The medium with native support showed a slight decrease in biomass at the end of the culture (8.58 g/L). The control group consistently demonstrates higher biomass in both variants, indicating that the addition of a loofah sponge confirmed the yeast immobilization and nesting within the sponge’s pores.

**Figure 8:**
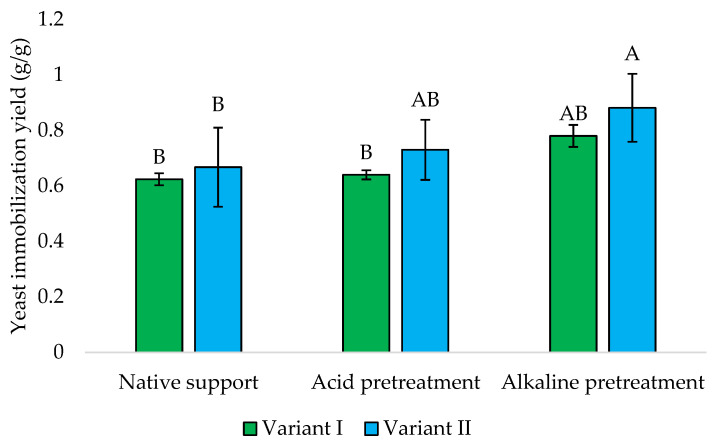
The yield of *Y. lipolytica* KKP 379 immobilization onto loofah sponge. Explanations: Variant I—1 g of loofah sponge was added at the beginning of culture; Variant II—1 g of loofah sponge was added after 48 h of culture. Error bars represent the standard deviation (SD) of three independent measurements. The values with the same uppercase letters (A, B) did not differ significantly (α = 0.05).

The immobilization of *Y. lipolytica* onto loofah sponge has been studied to evaluate its effectiveness in binding yeast cells under different conditions. The yields of this process are presented in Figure 8. The obtained results suggest that the timing of loofah sponge addition significantly affects the immobilization efficiency of *Y. lipolytica*, while later addition (Variant II) generally yielded higher biomass content in the sponge across all treatments. This could be attributed to initial yeast growth dynamics before immobilization, allowing for a more developed population to interact with the sponge surface effectively.

The alkaline pretreatment yielded the highest values, i.e., 0.78 and 0.88 g/g, respectively, which indicated that alkaline conditions may enhance the binding capacity of the loofah sponge for yeast cells, mainly when introduced later in the cultivation process.

He et al. [31] found that the immobilization yield of *Rhizopus oryzae* onto the loofah sponge depends on the initial mass of the sponge used. The study observed a decrease in immobilization yield with an increase in the amount of sponge used. This was attributed to a growth problem caused by excessive amounts of sponge. This fungus, when immobilized on a loofah sponge, reached a concentration of 1.40 g/g of loofah sponge particles [31]. Baloch et al. [32] optimized the lipase-catalyzed production of biodiesel by immobilization the cells of the yeast-like fungus *Magnusiomyces capitatus* A4C onto different supports. *M. capitatus* cells immobilized onto a loofah sponge exhibited significant hydrolytic activity of 12.7 U/mL and a cell-loading capacity of 0.61 g/g. Then, immobilization onto scouring pads allowed for lower hydrolytic and cell-loading values, i.e., 10.2 U/mL and 0.53 g/g, and in the case of polyurethane sponge a higher cell-loading capacity of 0.64 g/g was observed along with lower hydrolytic activity of 7.3 U/mL. According to Wojcieszyńska et al. [33], various factors, including temperature, pH, incubation time, and the initial optical density of the culture, played crucial roles in developing a robust biofilm of *Stenotrophomonas maltophilia* KB2 in the loofah sponge and its final application in naproxen biodegradation.

**Figure 9:**
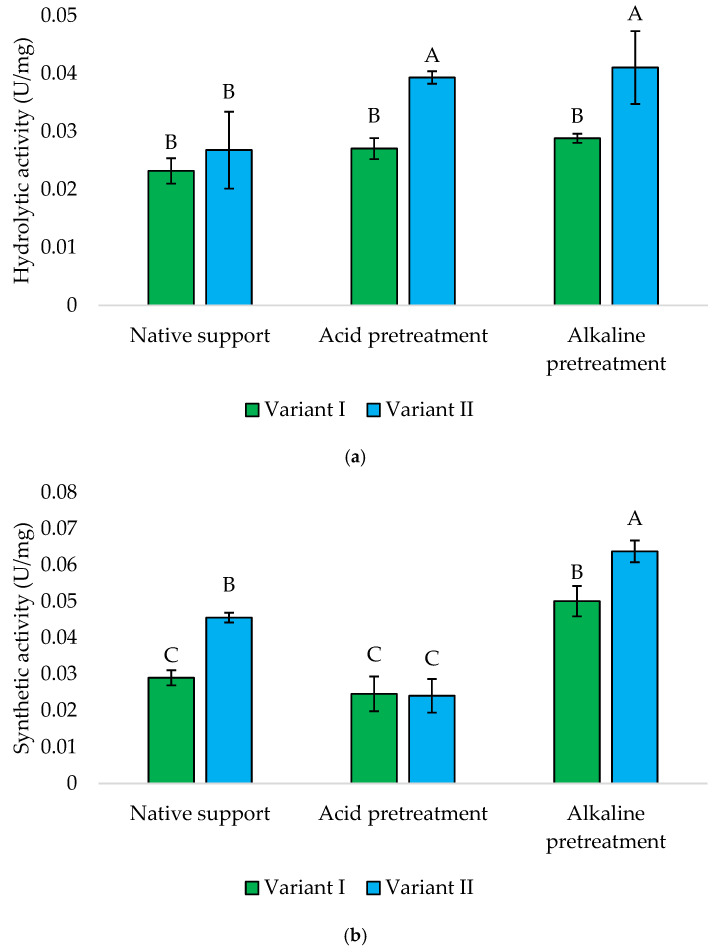
Hydrolytic (**a**) and synthetic (**b**) activities of *Y. lipolytica* KKP 379 immobilized onto a loofah sponge. Explanations: Variant I—1 g of loofah sponge was added at the beginning of culture; Variant II—1 g of loofah sponge was added after 48 h of culture. Error bars represent the standard deviation (SD) of three independent measurements. The values with the same uppercase letters (A–C) did not differ significantly (α = 0.05).

The immobilized cells onto the loofah sponge have been effectively utilized for treating wastewater containing toxic metals, dyes, and chlorinated compounds. Additionally, this technology has been employed to produce various secondary metabolites such as ethanol, organic acids, and enzymes [25]. This study also demonstrates the possibility of obtaining whole-cell biocatalysts immobilized on low-cost carriers. Both hydrolytic and synthetic activities of *Y. lipolytica* yeasts are presented in Figure 9. Similarly to the results described above, the proper approach to immobilization was crucial. Later addition of sponge (Variant II) generally yielded higher enzymatic activities across all treatments. In the case of hydrolytic activity, alkaline, and acidic pretreatments resulted in the highest hydrolytic activity, with Variant II outperforming Variant I, with activities of 0.041 and 0.039 U/mg, respectively.

The synthetic activities of *Y. lipolytica* KKP 379 immobilized onto the alkaline-pretreated loofah sponge were the highest observed within all the tested biocatalysts. Once again, Variant II exhibited significantly higher synthetic activity compared to Variant I (0.064 vs. 0.050 U/mg). In contrast to hydrolytic activity, the results for yeast immobilized onto acid-pretreated material were remarkably lower (±0.025 U/mg) compared to alkaline and native supports.

Several studies utilized the loofah sponge to immobilize yeast, fungi, and bacteria for producing lipases and applying the obtained formulations as whole-cell catalysts in various esterification, transesterification, and epoxidation reactions [31,34,35,36]. Lipase from *Burkholderia cepacia* was immobilized on starch film, agar gel, and loofa sponge to produce 1-octyl acetate, 1-pentyl octanoate, and monoepoxide from β-caryophyllene. The immobilized lipases were able to function in various organic solvents and ionic liquids. Using a loofah sponge as a carrier allowed for the production of over 99% of 1-octyl acetate in methyl tert-butyl ether and 1-pentyl octanoate in hexane as the solvents [35]. In another study, the immobilized *R. oryzae* cells were utilized to study biodiesel production. The whole-cell biocatalyst demonstrated hydrolytic and esterification activities, which increased with cell immobilization. At the same time, the reduced release of lipase into the culture medium indicated that the loofah sponge positively influenced cell immobilization [36].

## 4. Conclusions

The study investigated several agri-food and waste materials differentiated by structure and origin as carriers for the immobilization of free enzyme proteins and microbial cells. The carriers were characterized in terms of morphology, and the most important factor was the structure of pores and microspaces enabling immobilization via adsorption. Considering the importance of the presence of functional groups in the polymers comprising the plant-derived carriers, an attempt was made to identify them by rapid instrumental methods like FTIR and analytical methods like dry combustion. In the described studies, it was confirmed that the effect of carrier pretreatment on immobilization performance depends on both process conditions and the carrier itself, and protocols cannot be directly transferred between matrices.

The positive effect of carrier pretreatment consisted mainly in the removal of some of the components of the complex matrix of agri-food materials or waste rather than in the modification of the functional groups of the polymers forming the carrier. Of the four car carriers tested, the effect of alkaline pretreatment for loofah sponge on the hydrolytic and synthetic activity of free lipases as well as whole cells of yeasts was observed. For this particular carrier, it was also possible to additionally observe a reduction in the number of carbonyl groups, which could affect the results. In addition, brewed yerba mate showed promising results concerning the synthetic activity of adsorbed enzymes. The high activity of lipase adsorbed on brewed yerba mate and alkaline pretreated loofah sponge could be related to lignin removal. A more detailed description of these changes requires in-depth research. It seems reasonable to pay special attention to the presence of cellulose and hemicellulose and its exposure in plant material. The findings underscore the potential of lignocellulosic materials from the agri-food industry as effective supports for enzyme immobilization, giving an example of circular economy application in food processing and waste management.

## Figures and Tables

**Table 1:** Elemental analysis of the supports used for the immobilization of Sustine^®^ 121.

Support		Percentage Content (%)		
	Pretreatment *	C	N	S
Pea Hull	–	41.26 ± 0.03 ^j^	1.25 ± 0.02 ^c^	0.065 ± 0.008 ^e^
	H	41.11 ± 0.10 ^j^	1.20 ± 0.07 ^cd^	0.062 ± 0.006 ^e^
	H + EtOH	41.10 ± 0.02 ^j^	1.15 ± 0.02 ^d^	0.064 ± 0.001 ^e^
Buckwheat Husk	–	46.48 ± 0.09 ^f^	0.58 ± 0.07 ^e^	0.026 ± 0.001 ^f^
	H	47.18 ± 0.12 ^e^	0.59 ± 0.01 ^e^	0.023 ± 0.006 ^f^
	H + EtOH	47.16 ± 0.16 ^e^	0.60 ± 0.02 ^e^	0.027 ± 0.001 ^f^
Yerba Mate Leaves	–	49.39 ± 0.10 ^b^	2.30 ± 0.01 ^b^	0.104 ± 0.003 ^d^
	B	50.84 ± 0.01 ^a^	2.37 ± 0.06 ^b^	0.128 ± 0.003 ^c^
	B + H	48.22 ± 0.09 ^c^	2.84 ± 0.04 ^a^	0.163 ± 0.001 ^a^
	B + H + EtOH	47.72 ± 0.07 ^d^	2.76 ± 0.01 ^a^	0.151 ± 0.001 ^b^
Loofah Sponge	–	44.33 ± 0.11 ^g^	0.57 ± 0.07 ^e^	0.023 ± 0.001 ^f^
	Acid	44.02 ± 0.10 ^h^	0.38 ± 0.06 ^f^	0.023 ± 0.007 ^f^
	Alkaline	43.26 ± 0.02 ^i^	0.42 ± 0.12 ^f^	0.019 ± 0.002 ^f^

* “–”—Native supports; H—hexane; EtOH—ethanol; B—brewing; the values with the same lowercase letter (a–j) in the column did not differ significantly (α = 0.05).

## Data Availability

The original contributions presented in the study are included in the article and supplementary materials, further inquiries can be directed to the corresponding author (A.F.).

## Supplementary Materials

The following supporting information can be downloaded at: https://www.mdpi.com/article/10.3390/foods13233759/s1, Figure S1. FTIR spectra of *Luffa cylindrica* sponge: Luf_H_2_O—untreated raw material (red line), Luf_CH_3_COOH—sponge treated with acetic acid (green line), Luf_NaOH—sponge treated with sodium hydroxide (blue line); Figure S2. FTIR spectra of *Luffa cylindrica* sponge with immobilized lipase: Luf_H1—untreated sponge (red line), Luf_C1—sponge treated with acetic acid (purple line), Luf_N1—sponge treated with sodium hydroxide (blue line); Figure S3. FTIR spectra of buckwheat husks: untreated (yellow line), after hexane treatment (green line), and after ethanol treatment (red line); Figure S4. FTIR spectra of buckwheat husks with immobilized lipase: N_L—untreated (green line), H_L—after hexane treatment (blue line), and HE_L—after ethanol treatment (red line); Figure S5. FTIR spectra of Yerba Mate leaves: untreated (dark purple line), brewed (red line), brewed after hexane treatment (light purple line), and after ethanol treatment (blue line); Figure S6. FTIR spectra of Yerba Mate with immobilized lipase: N_L—untreated (dark blue line), P_L—brewed (green line), PH_L—brewed after hexane treatment (red line), and PHE_L—brewed after ethanol treatment (light blue line); Figure S7. FTIR spectra of pea hulls: untreated (blue line), after hexane (red line), and after ethanol treatment (purple line); Figure S8. FTIR spectra of pea hulls with immobilized lipase: N_L—untreated (red line), H_L—after hexane treatment (blue line), and HE_L—after ethanol treatment (purple line).
