# Supplementary material for: Agri-Food and Food Waste Lignocellulosic Materials for Lipase Immobilization as a Sustainable Source of Enzyme Support—A Comparative Study
Source: Foods. 2024 Nov 24;13(23):3759. doi: 10.3390/foods13233759 (PMC11640310; doi:10.3390/foods13233759)
Supplement: Supplementary file 1 [file foods-13-03759-s001.zip › foods-3324811-supplementary.pdf]

Article

Lignocellulosic Materials for Lipase Immobilization as a Sustainable Source of Enzyme Support – A Comparative Study

Bartłomiej Zieniuk, Jolanta Małajowicz, Karina Jasińska, Katarzyna Wierzchowska, Şuheda Uğur, and Agata Fabiszewska*

Department of Chemistry, Institute of Food Sciences, Warsaw University of Life Sciences-SGGW, Nowoursynowska 159c, 02-776 Warsaw, Poland; bartlomiej_zieniuk@sggw.edu.pl (B.Z.), jolanta_malajowicz@sggw.edu.pl (J.M.), karina_jasinska@sggw.edu.pl (K.J.), katarzyna_wierzchowska1@sggw.edu.pl (K.W.), suheda_ugur@sggw.edu.pl

* Correspondence: agata_fabiszewska@sggw.edu.pl (A.F.)

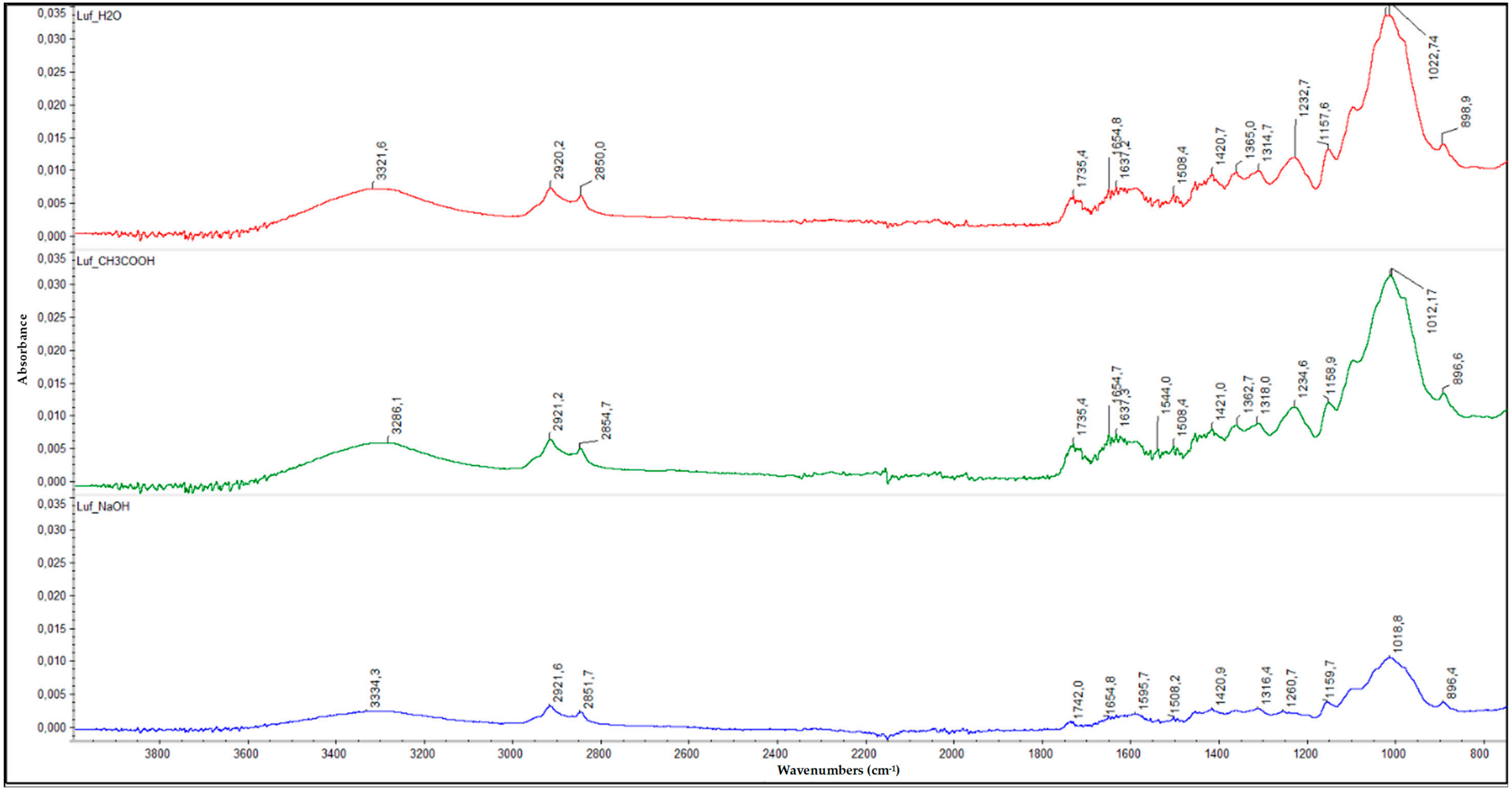

Figure S1. FTIR spectra of *Luffa cylindrica* sponge: Luf_H2O – untreated raw material (red line), Luf_CH3COOH – sponge treated with acetic acid (green line), Luf_NaOH – sponge treated with sodium hydroxide (blue line)**.**

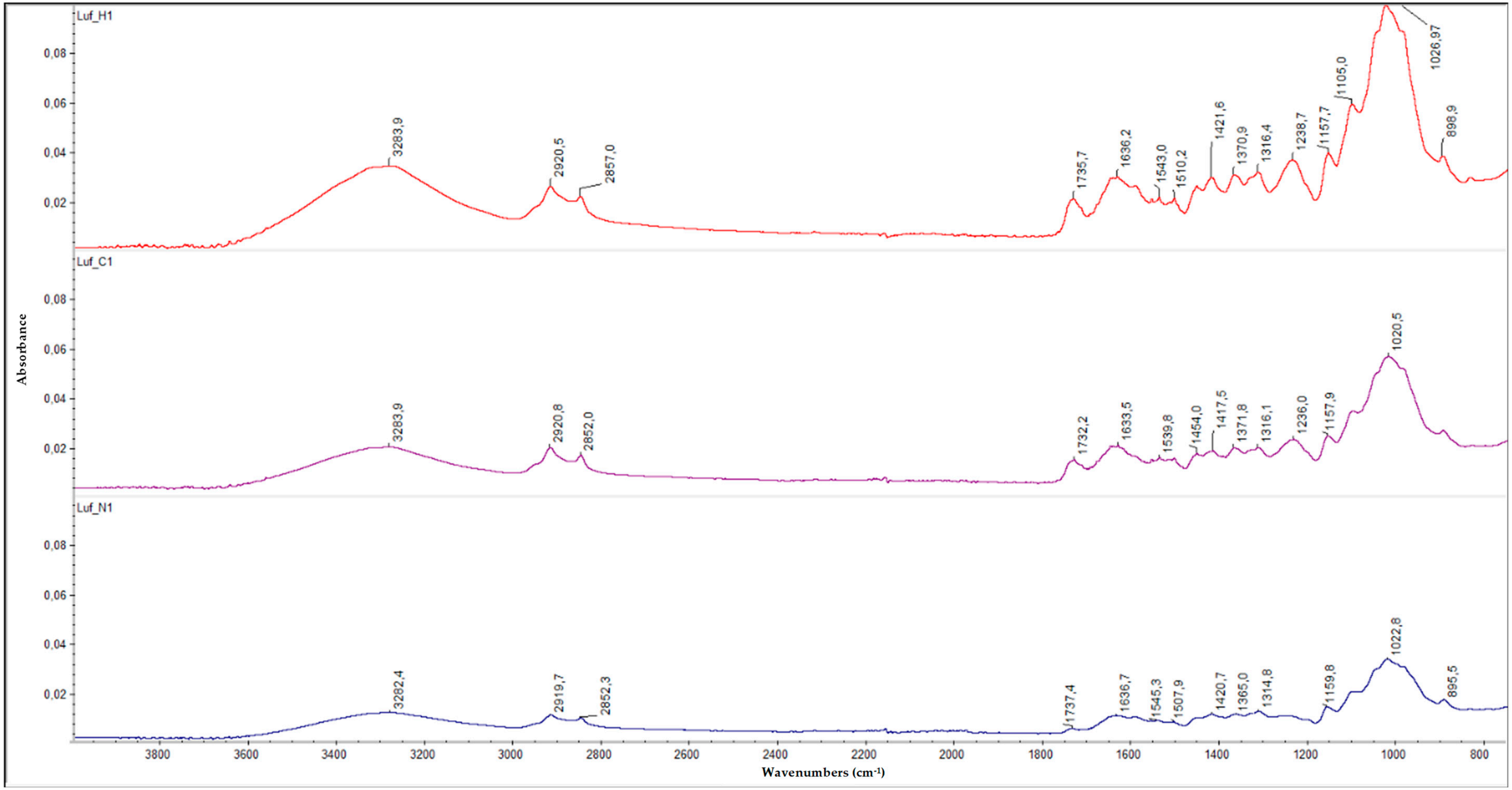

Figure S2. FTIR spectra of *Luffa cylindrica* sponge with immobilized lipase: Luf_H1 – untreated sponge (red line), Luf_C1 – sponge treated with acetic acid (purple line), Luf_N1 – sponge treated with sodium hydroxide (blue line).

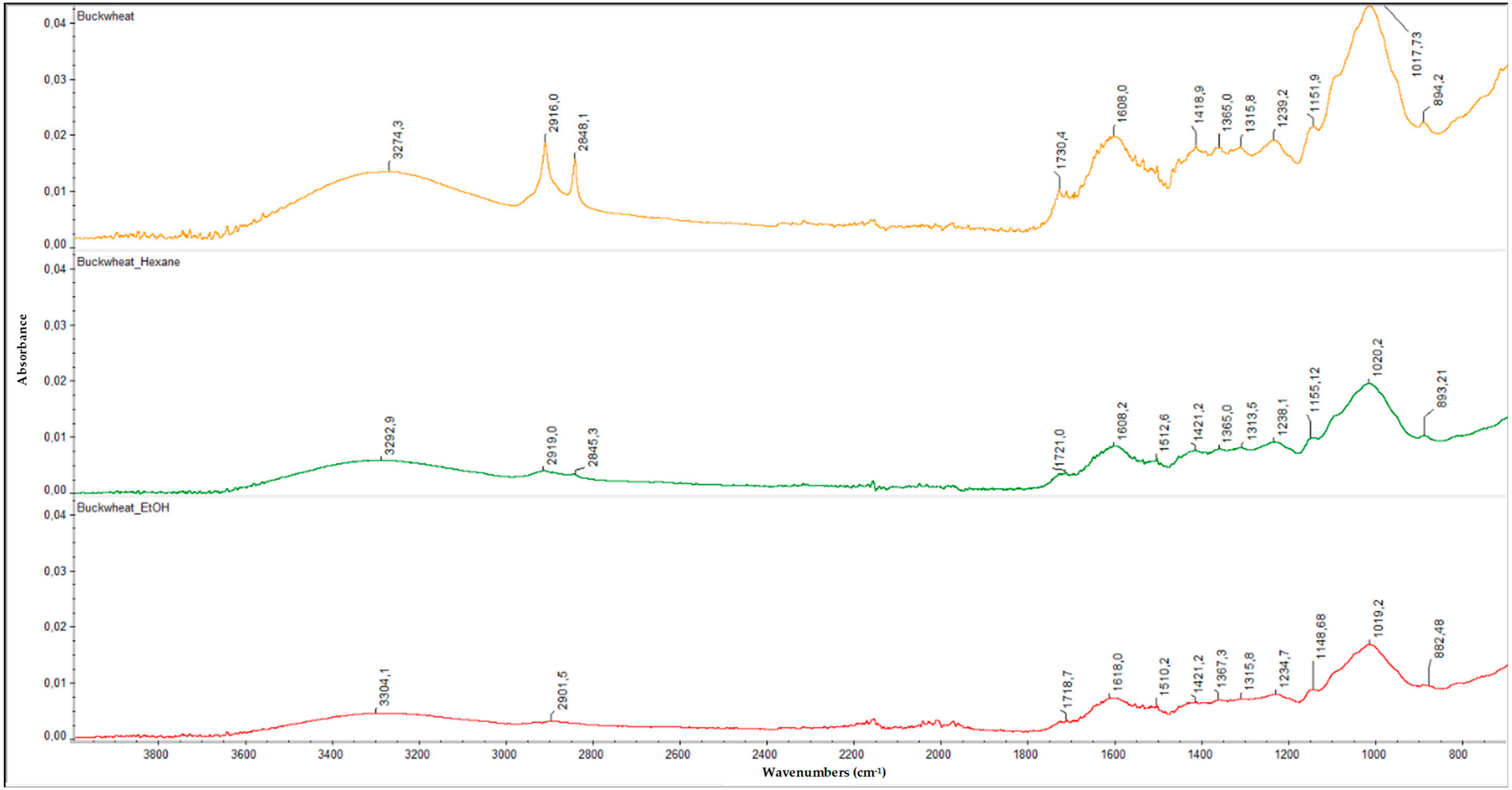

Figure S3. FTIR spectra of buckwheat husks: untreated (yellow line), after hexane treatment (green line), and after ethanol treatment (red line).

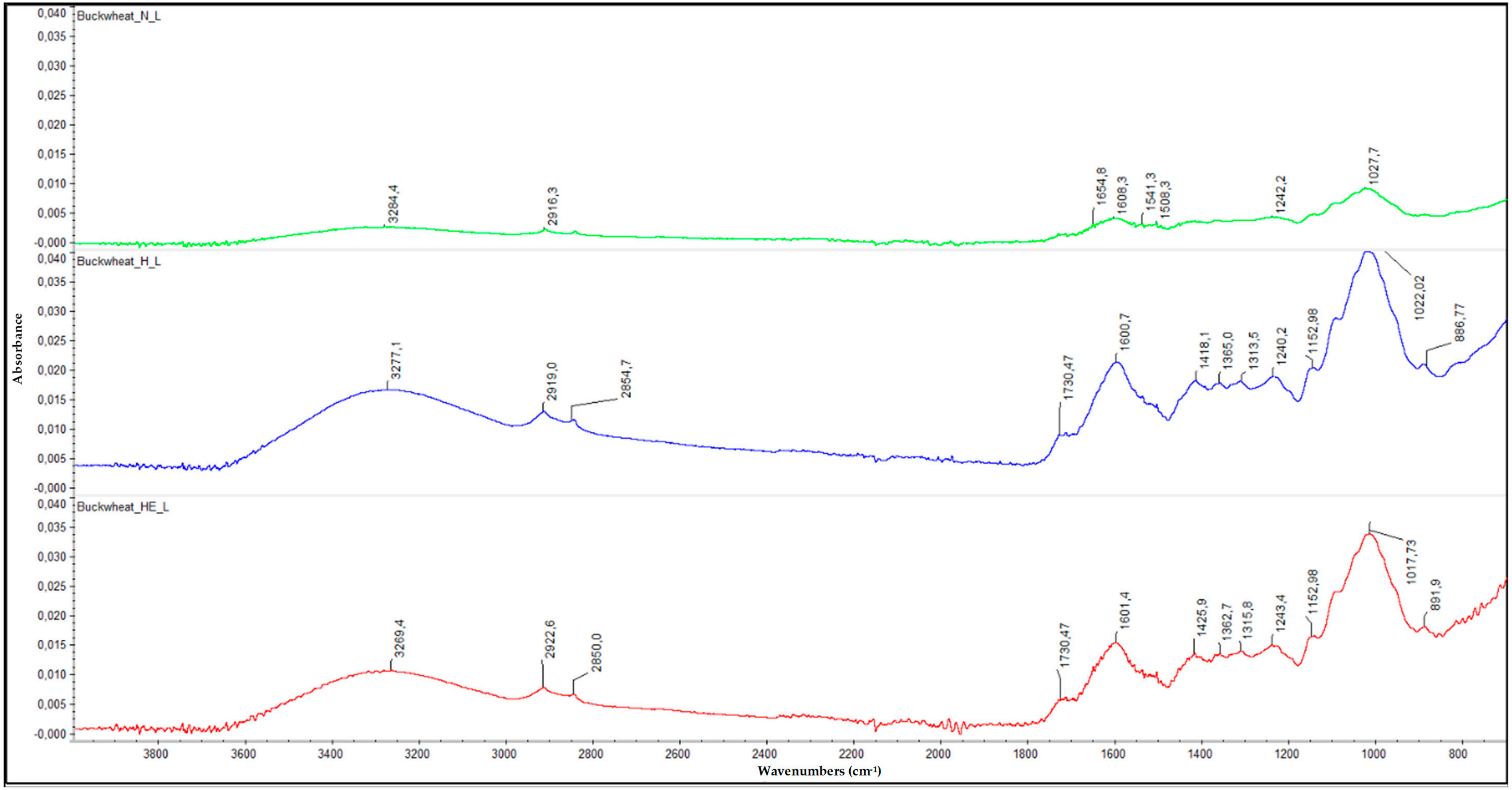

Figure S4. FTIR spectra of buckwheat husks with immobilized lipase: N_L – untreated (green line), H_L - after hexane treatment (blue line), and HE_L - after ethanol treatment (red line).

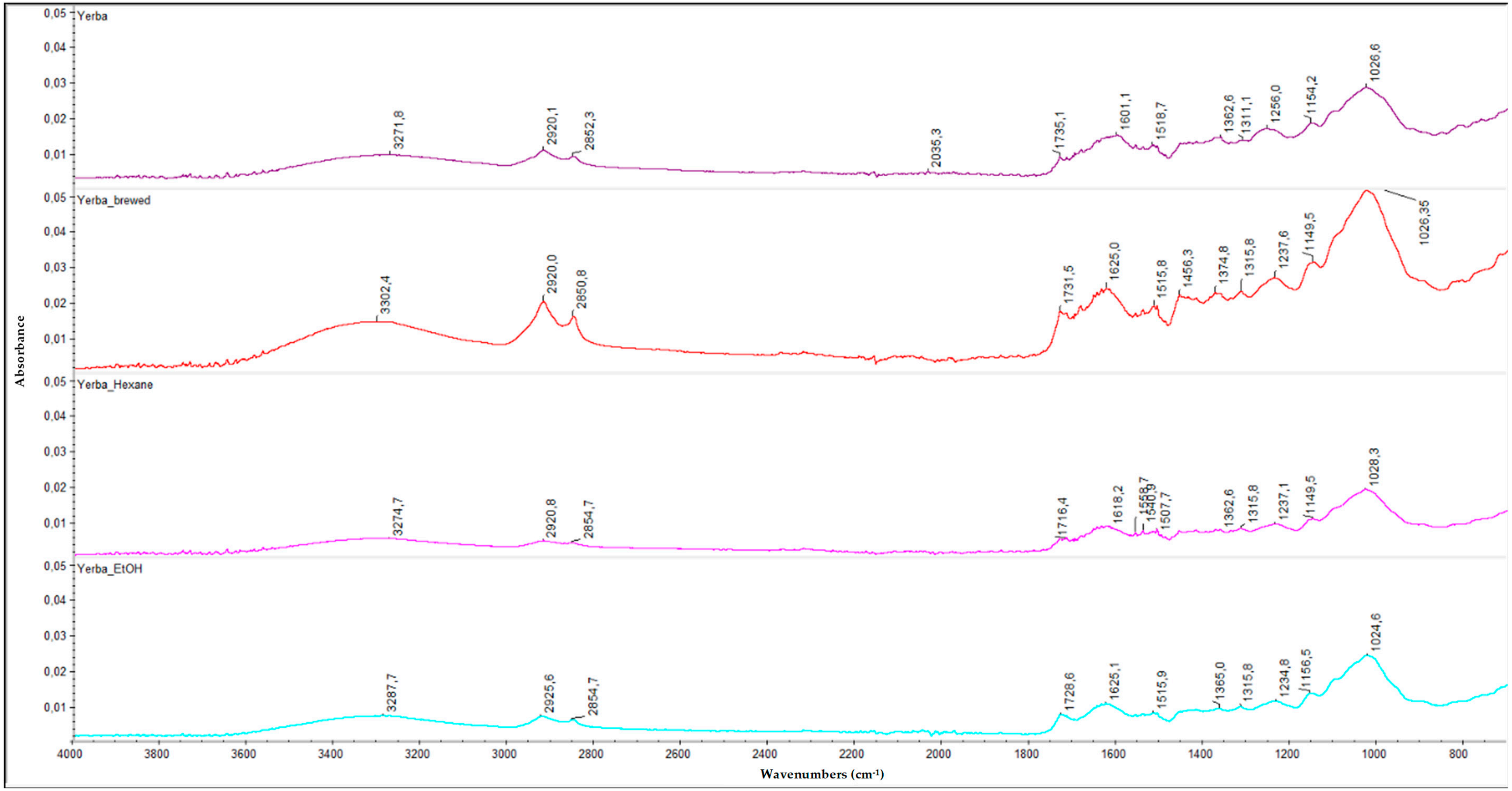

Figure S5. FTIR spectra of yerba mate leaves: untreated (dark purple line), brewed (red line), brewed after hexane treatment (light purple line), and after ethanol treatment (blue line).

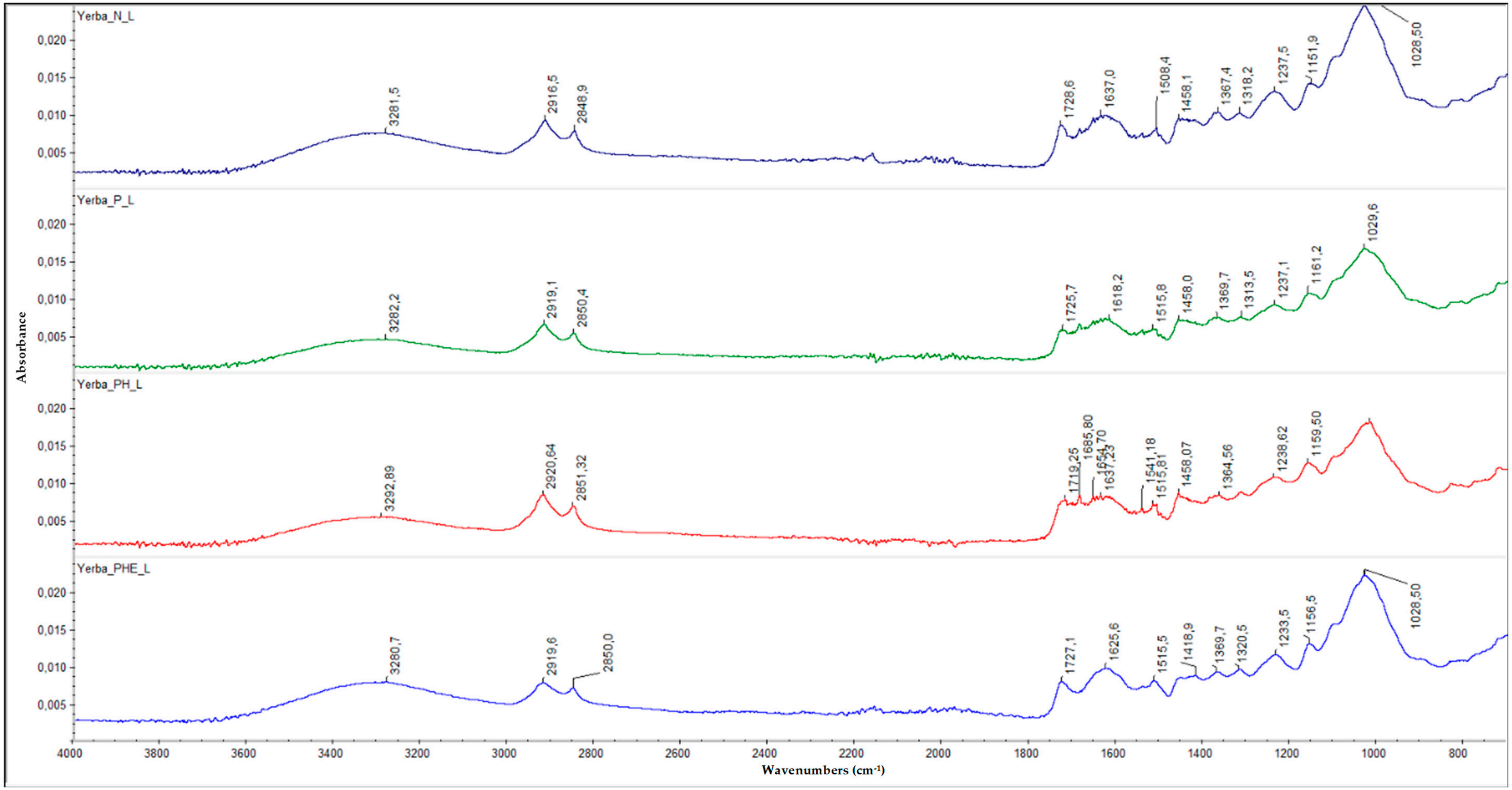

Figure S6. FTIR spectra of yerba mate with immobilized lipase: N_L – untreated (dark blue line), P_L – brewed (green line), PH_L – brewed after hexane treatment (red line), and PHE_L – brewed after ethanol treatment (light blue line).

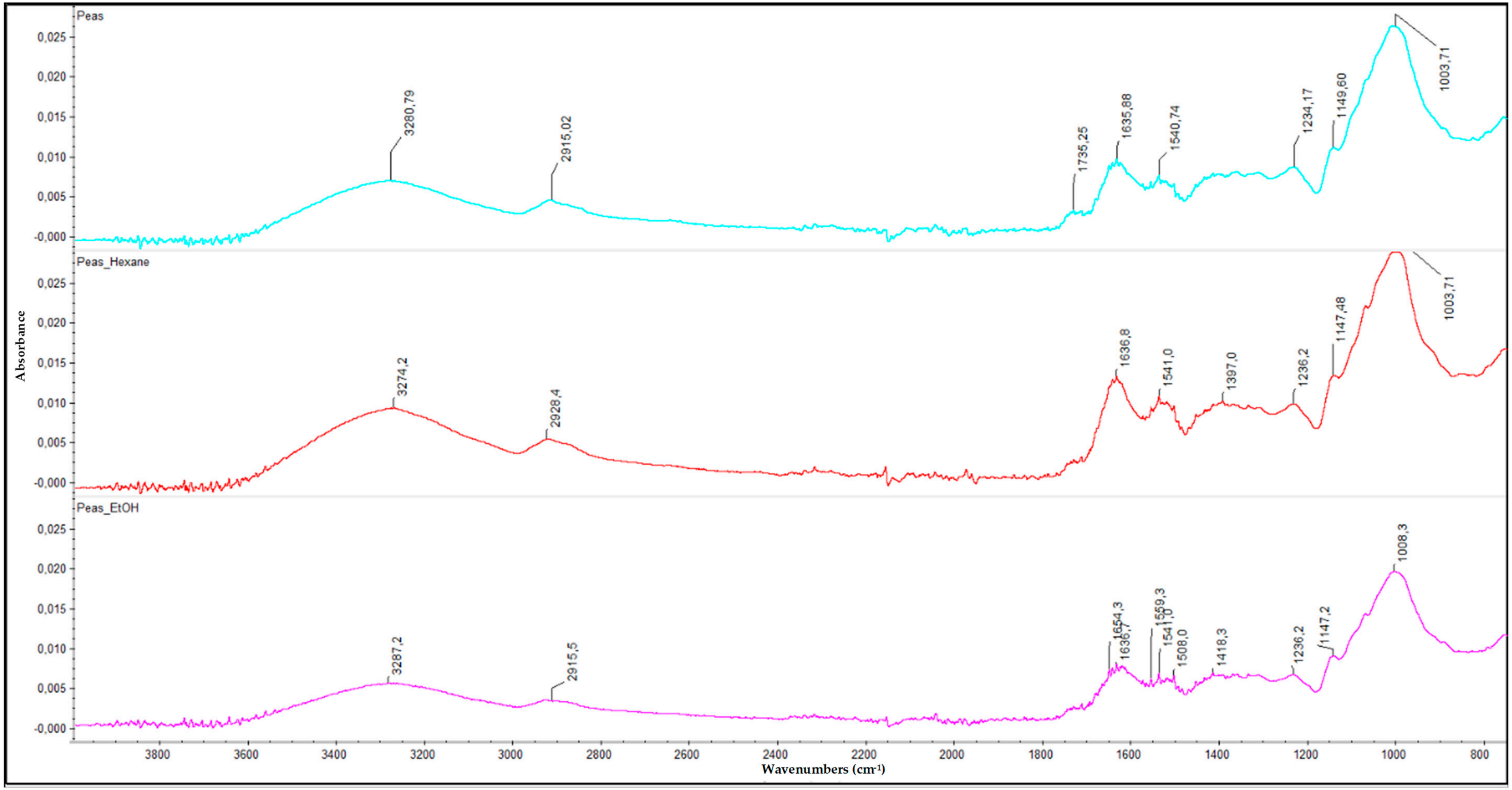

Figure S7. FTIR spectra of pea hulls: untreated (blue line), after hexane (red line), and after ethanol treatment (purple line).

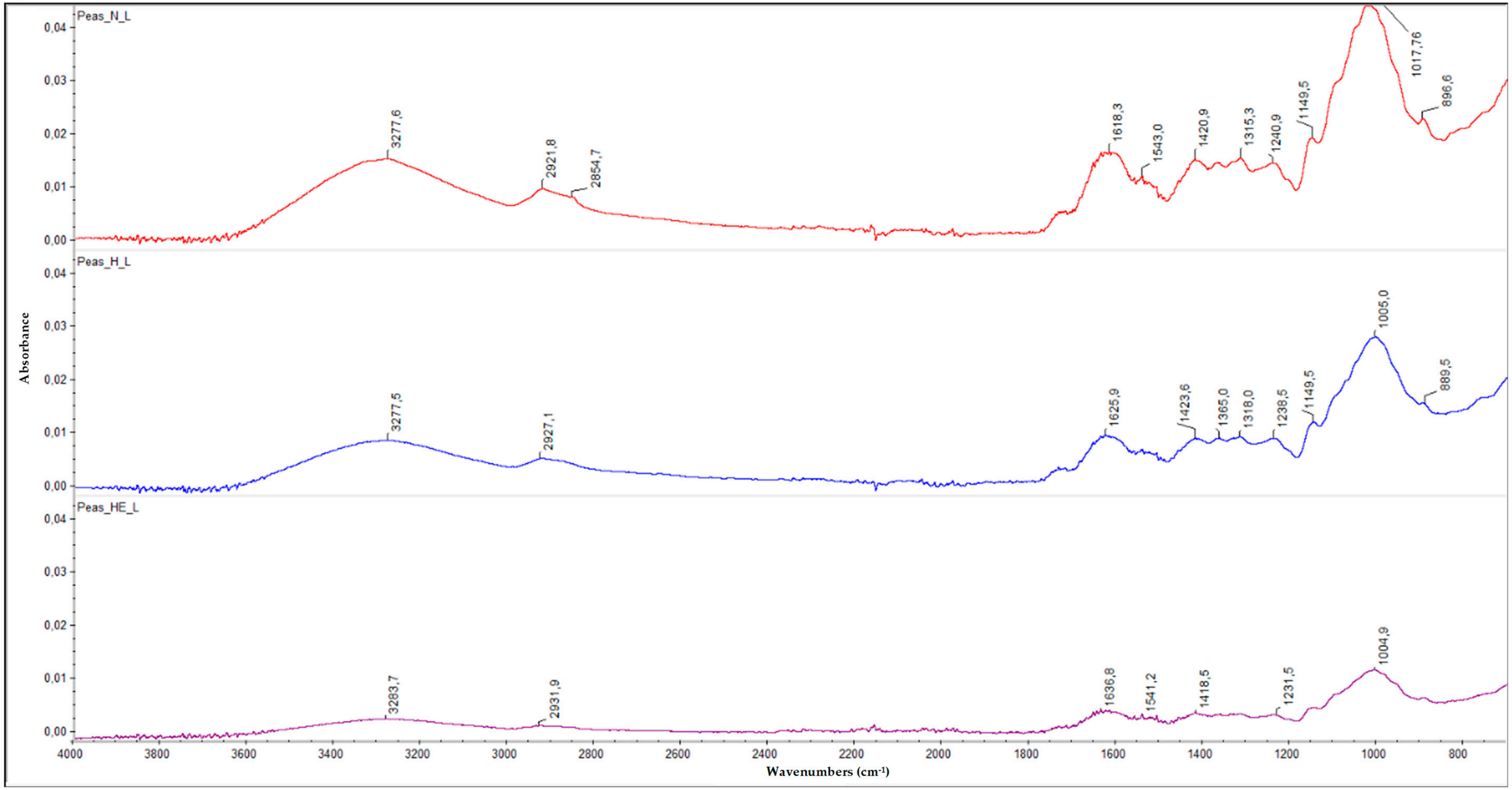

Figure S8. FTIR spectra of pea hulls with immobilized lipase: N_L – untreated (red line), H_L – after hexane treatment (blue line), and HE_L – after ethanol treatment (purple line).
